# Pharmacokinetics of Panaxynol in Mice

**DOI:** 10.26502/jcsct.5079059

**Published:** 2020-06-01

**Authors:** Hossam Tashkandi, Anusha Chaparala, Sean Peng, Mitzi Nagarkatti, Prakash Nagarkatti, Alexander A. Chumanevich, Lorne J. Hofseth

**Affiliations:** 1 Drug Discovery and Biomedical Sciences, College of Pharmacy, University of South Carolina, Columbia, SC, USA; 2 OB/GYN, Feinberg School of Medicine, Northwestern University, Chicago, IL, USA; 3 Touchstone Biosciences, Plymouth Meeting, PA, USA; 4 Pathology, Microbiology, and Immunology, School of Medicine, University of South Carolina, Columbia, SC 29208, USA

**Keywords:** Panaxynol, Falcarinol, Pharmacokinetics, Ginseng, Mice, Half-life, Bioavailability

## Abstract

The purpose of our study is to explore the pharmacokinetic parameters of panaxynol (PA) and understand its potential and dosage used in pre-clinical animal models. For *in vitro* analysis,5 μM of PA was added to liver microsomes of mouse and human species. Nicotinamide adenine dinucleotide phosphate was added to initiate enzyme reaction except for the negative control. Liquid chromatography with tandem mass spectrometry (LC-MS/MS) analysis was used to measure concentrations. For *in vivo* studies, CD-1 mice were treated with PA by intravenous (IV) injection or oral administration (PO). Concentrations of PA were measured in plasma and tissue using LC-MS/MS. Pharmacokinetic parameters were obtained using non-compartmental analysis. Area under the curve concentration versus time was calculated using a linear trapezoidal model.*In vitro*, PA’s half-life is 21.4 min and 48.1 min in mouse and human liver microsomes, respectively. *In vivo*, PA has a half-life of 1.5 hr when IV-injected, and 5.9 hr when administered via PO, with a moderate bioavailability of 50.4%. Mice show no signs of toxicity up to 300 mg/kg PO. PA concentrations were highest in colon tissue 2 hr post-treatment at 486 ng/g of colon tissue.PA’s pharmacokinetic properties and low toxicity point to the safety and compatibility of PA with mice.

## 1. Introduction

Native Americans have used ginseng for medicinal purposes for over a millennium. Ginseng is part of the Araliaceae family in the genus *Panax.* One of the most common types of ginseng is *P. quinquefolius* (American ginseng [AG]) [1, 2]. Ginseng has been shown to be a chemopreventive agent in the stomach, liver, pharynx, pancreatic, and colon cancers [3, 4]. It can also improve mental performance and prevent detrimental endpoints that are associated with inflammation and diseases such as diabetes, influenza, and cardiovascular disease [5].

Our lab has shown that AG is able to prevent colitis in mice [6, 7]. Since AG is a slurry of many compounds, we set out to determine which particular compound within AG is responsible for the suppression of colitis and prevention of colon cancer. To that end, bioassay-guided fractionation was used to delineate the active component of AG against colitis. We discovered that the hexane fraction of AG (HAG) is particularly potent at suppressing the induction of inducible nitric oxide synthetase *in vitro* and inflammation *in vivo*. Further, we were also able to show that HAG suppresses colitis associated colon cancer in azoxymethane/dextran sulfate sodium (DSS) mouse model [8].

Through preparative, reverse-phase high-performance liquid chromatography and ultraviolet/diode array detection, we have been able to further sub-fractionate HAG and identify an abundant molecule with exceptional potency against inflammatory endpoints. This molecule, called panaxynol (PA; also known as falcarinol), is a polyacetylene that is found in many plants such as carrots, celery, and parsnips [9, 10]. Originally, PA was discovered first by Takahashi *et al.* in *Panaxy ginseng C. A. Meyer* (Asian ginseng) and, independently, by Bohlmann *et al.* in *Falcaria vulgaris* [11, 12]. Furthermore, it has been shown to have anti-cancerous properties [13, 14]. However, research is lacking regarding the response of the body to PA. Finding out the half-life, bioavailability, toxicity, and other pharmacokinetic (PK) properties would help us understand how the body may interact with PA. Therefore, this paper focuses on exploring the PK properties of PA in a mouse model.

## 2. Materials and Methods

### 2.1 Microsome metabolism assay

Microsomal metabolism experiment was previously described in 1994 [15]. Liver microsomes are obtained from BioIVT for both human and mouse (Cat# X008067, InVitroCYP M-Class 50-D Human Liver Microsomes [Mixed Gender]; Cat# M00501, Male ICR/CD-1 Mouse Liver Microsomes). For each test compound, samples at a final concentration of 5 μM were prepared in 25 mM potassium phosphate buffer with liver microsomes of each species at a final concentration of 0.5 mg/ml. The enzyme reaction was initiated by the addition of NADPH reagent at a final concentration of 1 mM. For negative control samples, NADPH reagent was not added. All samples were incubated at 37°C on a 50 RPM orbital shaker, and aliquots were collected at predetermined time points (0, 5, 10, 15, 30, and 60 min). Samples were precipitated with three volumes of acetonitrile containing propranolol as internal standards and centrifuged for 10 min at 2000 g before LC-MS/MS analysis of the supernatant solutions. Percent parent compound remaining was determined relative to 0 min incubation samples, from which the elimination half-life was calculated based on the natural log of percent compound remaining vs. time plot. The following parameters were calculated to estimate the compounds *in vitro* metabolic stability: 1. C_mp_ = concentration of microsomal proteins (mg/ml); 2. t_1/2_ = the half-life (min), where t_1/2_ is equal to 0.693/slope.; 3. CL_int_ = the intrinsic hepatic clearance (μl/min/mg), where CL_int_ is equal to 0.693 / (t_1/2_ × C_mp_).

### 2.2 Mouse housing conditions

Animal housing protocol was similar to a previously published protocol with slight modifications [16]. Animals (mouse) were fed a Teklad Global 16% protein rodent diet and housed in individual cages on a 12 hr light and 12 hr dark cycle with room temperature maintained at 22 ± 3°C and relative humidity at 50 ± 20%. Animals were fasted overnight before dosing, with food returned after the 6 hr blood samples were obtained. Water was provided ad libitum throughout the study. All animal experiments were approved by the Institutional Animal Care and Use Committee at the University of South Carolina and were in accordance with the National Institute of Health guidelines for the treatment of animals.

### 2.3 PA formulation

For 5 mg/kg IV and 20 mg/kg PO experiments: 5% dimethylacetamide + 20% ethanol + 40% polyethylene glycol 300 + 35% water (solution). For 100, 200, and 300 mg/kg PO experiments: 1% carboxymethyl cellulose in water (suspension).

### 2.4 *In Vivo* pharmacokinetic procedures

Animals were dosed via gavage needle for oral administration at 20, 100, 200, and 300 mg/kg (PO) or via tail vein injection for intravenous (IV) administration at 5 mg/kg. Serial blood sampling was used to extract all blood samples (30–50 μl per sample). Blood samples were taken via saphenous vein at 5, 15, and 30 min and 1, 2, 4, 6, 8, and 24 h after dosing and processed for analysis. Indicated tissues were examined for PA content at the end of the study. All samples were stored at −70°C until analysis for side-by-side comparison.

### 2.5 Bioanalysis of *In Vivo* pharmacokinetic plasma samples

Plasma bioanalysis samples were prepared based on previously published methods [17]. In short, three volumes of acetonitrile containing internal standard were added to one volume of plasma to precipitate proteins. Samples were centrifuged (3000 g for 10 min) and supernatants removed for analysis by LC-MS/MS. Calibration standards and quality controls were made by preparation of a 1 mg/ml stock solution in methanol and subsequently a series of working solutions in methanol : water (1/:1, v/v) which were spiked into blank plasma to yield a series of calibration standard samples in the range of 10 ng/ml to 10 μg/ml and quality control samples at three concentration levels (low, middle, and high). All incurred PK plasma samples were treated identically to the calibration standards and quality control samples. LC-MS/MS analysis was performed utilizing multiple reaction monitoring for detection of characteristic ions for each drug candidate, additional related analytes, and the internal standard.

### 2.6 Bioanalysis of *In Vivo* pharmacokinetic tissue samples

The bioanalysis samples were prepared based on previously published methods [17]. Briefly, tissue samples were prepared following euthanasia 24 hr post treatment. Three volumes of PBS buffer (pH 7.4) were added to one volume of each tissue sample, then homogenized to obtain each tissue homogenate sample. Subsequently, three volumes of acetonitrile containing internal standard were added to one volume of each tissue homogenate, the mixture vortexed, centrifuged (3000 g for 10 min), and supernatant removed for analysis by LC-MS/MS. Calibration standards were made by preparation of a 1 mg/ml stock solution and subsequently a series of working solutions in methanol : water (1/:1, v/v) which were spiked into blank tissue homogenate to yield a series of calibration standard samples in the range of 10 μg/ml to 10 μg/ml. All incurred tissue samples were treated identically to the calibration standards. LC-MS/MS analysis was performed, and characteristic ions detected for each drug candidate.

### 2.7 Pharmacokinetic data analysis

The data analysis was based on the plasma concentrations, which were measured as described above to determine a concentration vs. time profile. The area under the plasma concentration vs. time curve was calculated using the linear trapezoidal method. Fitting of the data to obtain pharmacokinetic parameters was carried out using non-compartmental analysis [18]. Tables were generated using Microsoft Word 365 ProPlus. Figures and analysis were generated in GraphPad Prism.

## 3. Results

### 3.1 Microsomal metabolism

**Figure 1:**
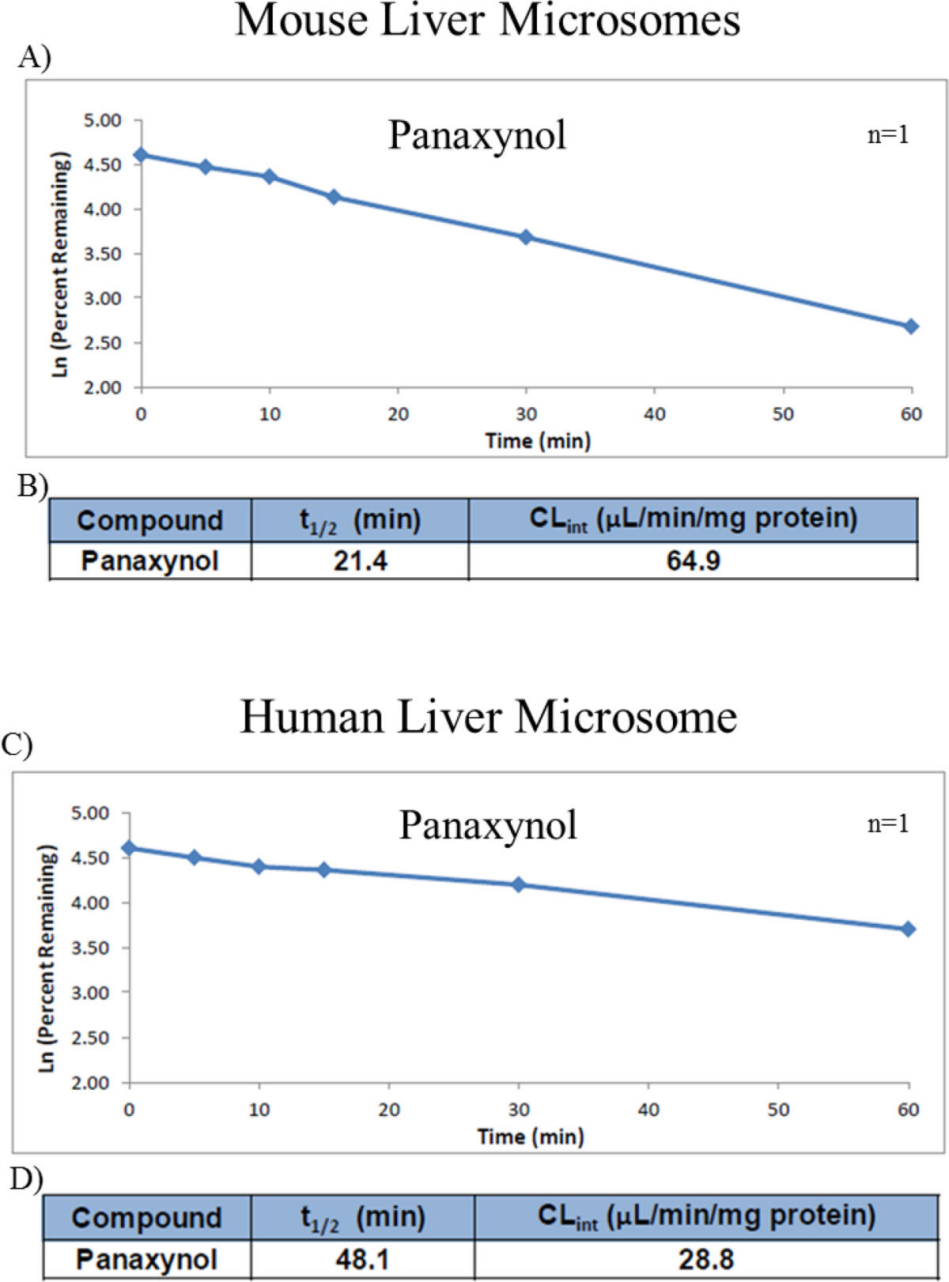
PA and liver microsome metabolism in both mouse and human microsomes (n=1). A and C are the graphs for the natural log of the percent remaining PA against time in minutes for both mouse and human liver microsomes respectively. B and D are the calculated half-life (t_1/2_) and intrinsic clearance (CL_int_) of PA from the plotted graph of mouse and liver microsomes, respectively.

A significant amount of any ingestible drug gets metabolized in the liver using a family of enzymes called cytochrome P450 [19]. For that reason, PA’s stability and resistance to liver metabolism was assessed by using enzyme-containing liver microsomes from mice and humans. In mouse microsomes, the half-life (t_1/2_) of PA was 21.4 min (Figure 1A and B). In addition, PA’s intrinsic hepatic clearance (CL_int_) was 64.9 μl/min/mg protein, which is considered to be high (Figure 1B). On the other hand, PA has a longer half-life in human microsomes, which was 48.1 min (Figure 1C and D). Finally, the intrinsic hepatic clearance of PA in human microsomes was less than in mouse microsomes (28.8 μl/min/mg protein) and is considered as moderate clearance (Figure 1D).

### 3.2 Pharmacokinetic values of PA in mice

**Figure 2:**
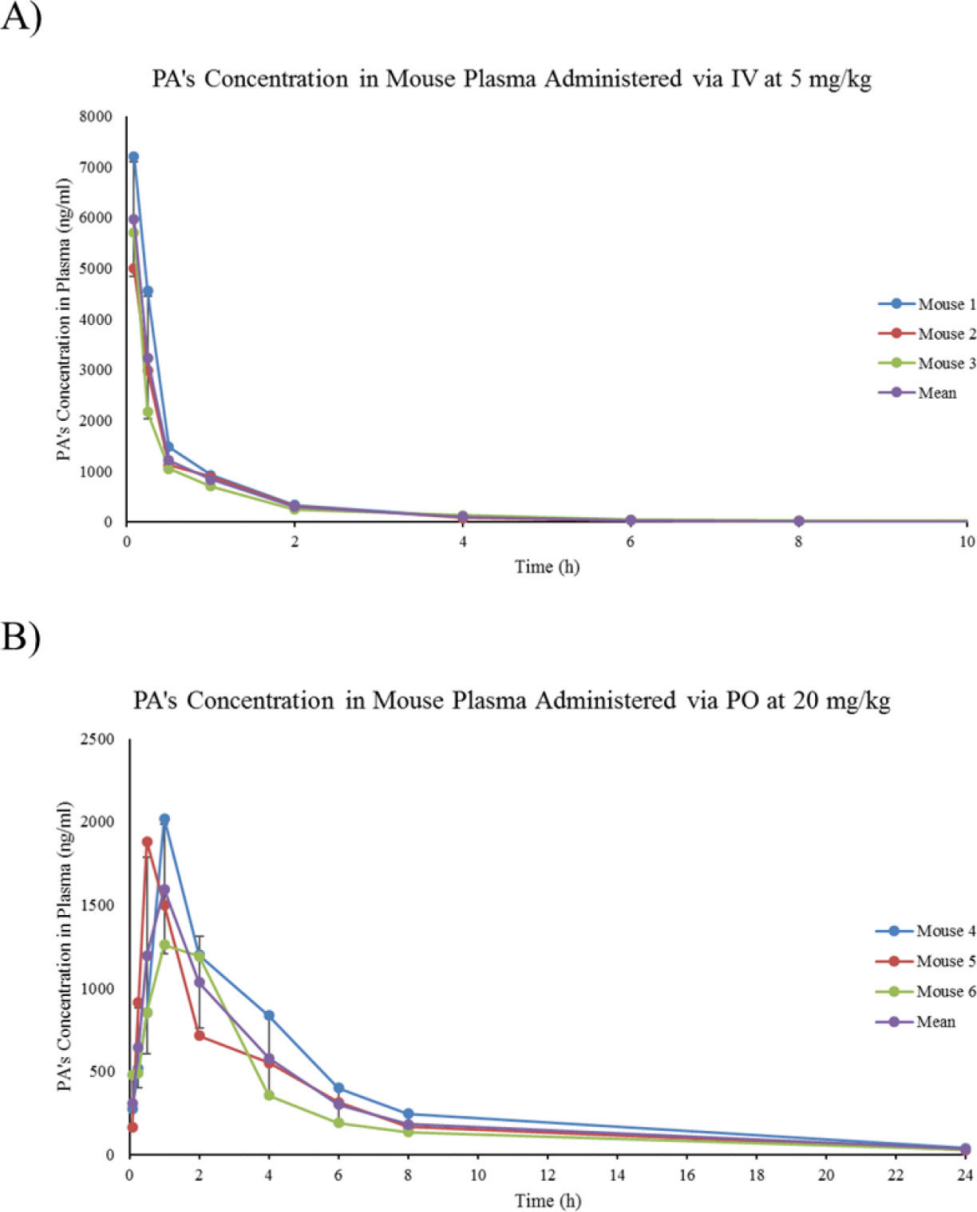
Graphs of PA treatment of mice (n=3). Each dot represents a time point at which PA’s concentration was measured in plasma. A) Linear graph of PA’s concentration in mouse plasma after injecting mice with PA via IV at a concentration of 5 mg/kg. B) Linear graph of PA’s concentration in mouse plasma after treating mice with PA via PO at a concentration of 20 mg/kg.

Using LC-MS/MS, plasma from mice (n=3) given PA by IV at 5 mg/kg and orally at 20 mg/kg was analyzed. These doses were chosen to ensure detectable levels of PA in plasma at all time points. Following the administration of PA by IV, PA reached its initial highest plasma concentration at 8.24 μg/ml, then declined in a multiphasic way (Figure 2A). On the other hand, plasma analysis of PA after PO administration showed the compound concentration reaching maximum peak of 1.72 μg/ml rapidly within 1 hr. Then, the plasma concentration declined in a multiphasic manner with a final measurable concentration of 32.2 ng/ml at 24 hr (Figure 2B).

PK parameter estimates of PA are shown in Table 1. IV injection of PA showed low half-life at 1.5 hr.In addition, the systemic clearance of PA via IV injection was low to moderate 23.5 ml/min/kg. The steady-state volume of distribution was moderate to high at 1.46 L/kg with a total systemic exposure of 3.61 hr*μg/ml. Taken together, this suggests low metabolism and high tissue distribution of PA *in vivo*. In addition, 20 mg/kg PO administration of PA showed a much longer (moderate) half-life of 5.89 hr. The steady-state volume of distribution was high at 15.86 L/kg with a total systemic exposure of 7.27 hr*μg/ml. Finally, PA had a bioavailability measured at 50.4% (Table 1).

**Figure 3:**
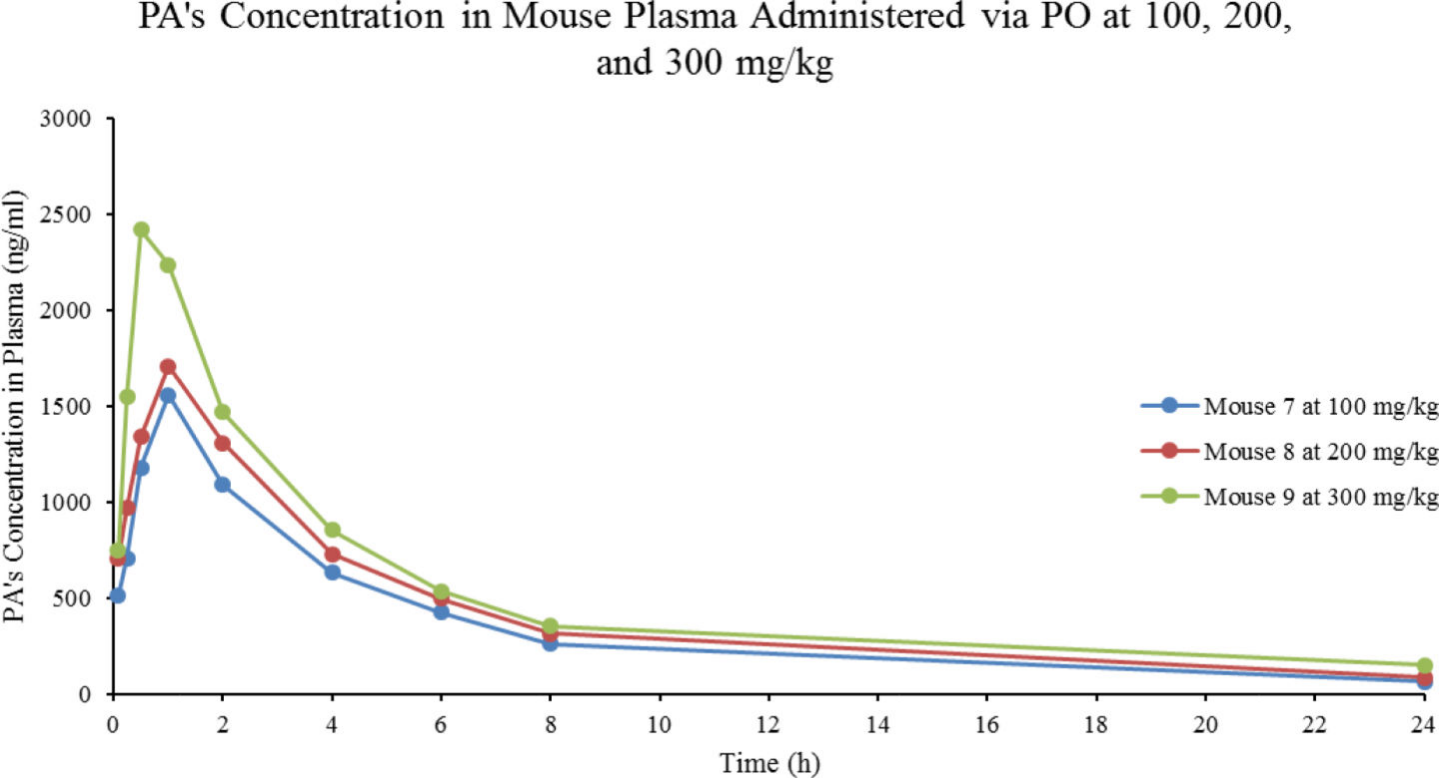
Linear graph of PA’s concentration in mouse plasma after treating mice with PA via PO at concentrations of 100/200/300 mg/kg. Each dot represents a time point at which PA’s concentration was measured in plasma.

Interestingly, mice treated with PA by PO up to 300 mg/kg showed no abnormal clinical symptoms. This indicates low toxicity potential by PA. After treating mice with 100, 200, and 300 mg/kg, the half-lives of PA were at 7.11, 7.67, and 9.15 hr, respectively (Table 2). PA rapidly reached a peak plasma concentration of 1.56, 1.71, and 2.42 μg/ml respectively within 1 hour. Following that, its plasma concentrations declined in a multiphasic manner with the last measurable concentrations of 65.1, 87.2, and 152 ng/ml at 24 hr, respectively (Figure 3). The total systemic exposure (AUC_inf_) was 9.10, 11.0, and 14.3 h*μg/ml with a low bioavailability (F) of 12.6, 7.61, and 6.59%, respectively (Table 2). Importantly, mice showed no abnormal clinical signs of toxicity in 24 hours of treating with PA.

Because PA was found in HAG, and that HAG was effective in treating DSS-induced colitis in mice [8], it was important to measure the concentration of PA in mouse colons. Colon tissues from mice treated via PO with PA at 20 mg/kg were harvested, then homogenized, and analyzed for PA concentration. PA concentration was highest in colon tissue 2 hr after treatment at 121 ng/ml of homogenized tissue solution (486 ng/g of colon tissue, Table 3). However, plasma concentration of PA reached a maximum at 1 hr. Finally, tissue/plasma concentration ratios show that PA was more concentrated in plasma as it gets absorbed but roughly 1/3^rd^ of PA stays in the colon (Table 3).

## 4. Discussion

After PA’s first discovery, it has been shown to reduce cancer proliferation and reduction of neoplastic lesions in rat colons [13, 14]. However, the PK properties of PA have not been described. Determining the PK factors of PA help us understand how the body responds to it. This helps in determining the correct dosage to reach PA effectiveness on various diseases. The first *in vitro* step taken to determine PA’s stability was to use human and mouse liver microsomes containing cytochrome P450. Drug metabolism occurs in the liver in a multiphasic manner. In phase 1, PA would have to endure catalysis by the cytochrome P450 group of enzymes to make the drug more soluble for easy elimination through the kidneys [20]. Our data suggests that PA has a high CL_int_ in mouse microsomes, whereas in human microsomes, PA has a moderate CL_int_. The difference between the two clearances is possibly determined by the types of cytochrome P450 available in mouse microsomes but not in human microsomes [21].

Mice treated with PA through IV showed low to moderate systemic clearance and moderate to high system distribution. This indicates that PA has low metabolism and high tissue distribution *in vivo*, and low plasma protein binding. Although PA had a short half-life (1.5 h) when given through IV, the half-life increases to a moderate 5.89 h when given using PO. Additionally, the steady-state volume of distribution and total system exposure are both higher in mice given PA through PO. Those may be explained due to the expected low absorption of PA as it is highly lipid soluble. In fact, as shown in the formulation, up to 5% of dimethylacetamide in the formulation was needed to dissolve PA in aqueous solution. In addition, the moderate bioavailability (50.4%) of PA is also due to the low metabolism and slow absorption. Interestingly, PA’s bioavailability was not maintained when the doses were increased up to 300mg/kg. It instead decreased further, supporting the likelihood of low absorption of PA or the different formulation needed due to the large amounts of PA. However, no signs of toxicity were observed in mice throughout the experiment. This is important in that we can safely change dosages for maximum efficacy in treating various diseases.

Understanding a drug PK at the site of action is important. For example, exploring drug distribution into the colorectum and genitals guided the development of pre-exposure prophylaxis for HIV infections [22]. Furthermore, because our research interest involves inflammatory bowel diseases and colorectal cancer, we found it important to determine the tissue distribution of PA in the colon. Unpublished data from our lab show PA effectiveness at reducing DSS-induced colitis in mice in as low of a dose as 0.1 mg/kg. Furthermore, other research has demonstrated prevention of inflammation in colon and colorectal neoplastic transformation in rats by PA [23]. Even though the results showed that PA is more concentrated in plasma, it is still detectable in the colon (up to 486 ng/g at a 20 mg/kg PO dose). This indicates that the tissue concentration, when PA is given at 0.1 mg/kg, was more than enough to achieve efficacious results against DSS-induced colitis.

Aside from the low absorption, PA has relatively favorable PK parameters. Further analysis of metabolites from PA and possible drug interactions would greatly enhance our understanding of its PK properties. An additional step to be taken is the determination of the pharmacodynamics of PA to further help in optimizing its binding and achieve better results. Another limitation is that we do not know the toxicity of PA over multiple doses in a day or over an extended period. The different percentage of PA enantiomers were not analyzed in the solution of PA given to the mice. PA’s interaction with other drugs are also poorly understood and is another limitation of this study. Given PA’s poor absorption, other anti-inflammatory drugs could decrease its absorption. Finally, we do not know PA’s PK values in some disease conditions. In particular, inflammatory bowel diseases increase the permeability of the gastrointestinal tract and there by affecting PA’s PK values.

Because PA is researched as a possible treatment for different diseases, it stands to believe that it is important to explore how an animal body responds to PA. With favorable PK results, PA is poised to be a safe molecule to further research *in vivo*. Finally, knowing the PK properties of PA will be important in comparing it against PA-derived molecules for treating diseases more effectively.

## Figures and Tables

**Table 1:** PK parameter calculated of PA treatments of mice (n=3) via IV at 5 mg/kg and PO at 20 mg/kg. CV% is the coefficient of variance percentage.

Parameter	Definition	IV	IV CV%	PO	PO CV%
t_1/2_ (hr)	Half-Life	1.5	6.38	5.89	18.8
CL_p_ (ml/min/kg)	Systemic Clearance	23.5	14.7	-	-
V_ss_ (L/kg)	Steady-State Volume of Distribution	1.46	26.2	15.86	22.78
F (%)	Bioavailability	-	-	50.4	21.4
AUC_inf_ (hr*ng/ml)	Total System Exposure	3609	16	7278	21.4

**Table 2:** Pharmacokinetics parameters calculated from mice treated with 100, 200, and 300 mg/kg of PA (n=1). t_1/2_ is the half-life of PA in mice. AUC_inf_ is the total system exposure. F represents the bioavailability of PA.

Concentration (mg/kg)	t1/2 (hr)	AUCinf (hr*ng/ml)	F (%)
100	7.11	9103	12.6
200	7.67	10981	7.61
300	9.15	14268	6.59

**Table 3:** PA concentration in colon tissue and plasma (n=3). BLQ stands for below limit of quantification (10 ng/ml). CV% is the coefficient of variance percentage. N/A stands for not applicable.

Time (hr)	Mean Plasma Concentration (ng/ml)	CV%	Mean Homogenized Concentration (ng/ml)	Mean Tissue Concentration (ng/g)	CV%	Mean Tissue/Plasma Concentration Ratio
0.5	890	22.1	BLQ	N/A	N/A	N/A
1	1333	25.8	56.9	228	42.7	0.256
2	1047	26.2	121	486	23.6	0.364
4	684	29.2	94.8	379	14.1	0.362

## Abbrevations

AG: American Ginseng
DSS: Dextran Sulfate Sodium
HAG: Hexane fraction of American Ginseng
PA: Panaxynol
PK: Pharmacokinetic
LC-MS/MS: Liquid Chromatography-Mass Spectrometry
IV: intravenous
PO: oral administration
